# Challenges of scale in assessing the risks of climate change for heritage

**DOI:** 10.1007/s00704-026-06386-6

**Published:** 2026-06-20

**Authors:** Scott Allan Orr, Shixin Zhao, Helen Thomas, Paloma Guzman, Courtney Hotchkiss, Erin Seekamp, Xiao Xiao, Chiara Aquilani, Ebrahim Ghaderpour, Josep Grau-Bové, Maya Ishizawa, Paul Lankester, Ionut Cristi Nicu, Michele Ortolani

**Affiliations:** 1 UCL Institute for Sustainable Heritage, 14 Upper Woburn Pl, London, WC1H 0NN UK; 2 Historic England, The Engine House, Fire Fly Avenue, Swindon, SN2 2EH UK; 3 Digital Archaeology Department, Norwegian Institute for Cultural Heritage Research (NIKU), Storgata 2, 0155, Oslo, Norway; 4 College of Natural Resources, North Carolina State University, Raleigh, NC 27695 USA; 5 School of Community Resources and Development, Arizona State University, 411 N. Central Avenue, Suite 550, Phoenix, AZ 85004 USA; 6 Department of Earth Sciences & CERI Research Centre, Sapienza University of Rome, Piazzale Aldo Moro 5, Rome, 00185 Italy; 7 International Centre for the Study of the Preservation and Restoration of Cultural Property (ICCROM), Via di San Michele 13, Rome, Italy; 8 English Heritage, The Engine House, Fire Fly Avenue, Swindon, SN2 2EH UK; 9 High North Department, Norwegian Institute for Cultural Heritage Research (NIKU), Fram Centre, Tromsø, N-9296 Norway; 10 Department of Physics, Sapienza University of Rome, Piazzale Aldo Moro 5, Rome, 00185 Italy

## Abstract

Heritage is a living process—our legacy from the past, including social and ecological systems, within which we live today and pass on to future generations. Assessing climate change risks is essential for understanding how hazards, exposure, vulnerability, and responses interact to produce impacts for heritage. These interactions operate across diverse spatial and temporal scales. Current approaches to risk assessment evaluate the scales of climate data, heritage processes, and governance decisions implicitly, leading to misalignments that limit how effectively risks are identified, interpreted, and managed. Here we show that these misalignments arise when observational, measurement, and operational scales diverge. The observational scale defines the boundary and timeframe of a risk assessment. The measurement scale concerns the resolutions of data used, from global climate models to site-level monitoring. The operational scale represents those of underlying processes, from short-term flooding to multi-decadal maintenance and knowledge transmission. When these scales diverge, mismatches obscure how climate change risks are understood and managed. These mismatches reveal not only technical challenges but also deeper divides between knowledge systems, institutional actions, and governance structures. A scale-aware approach can help translate risk assessments into effective actions, aligning data, processes, and responsibilities.

## Introduction

The management of heritage in the context of climate change prompts a rethinking of how we conceptualise risk, resilience, and responsibility. Conservation, understood as the management of change, requires responses to climate change through preventive measures, adaptation strategies, and the sharing of knowledge and best practices across all levels of governance and society (UNESCO World Heritage Centre, 2006). In this perspective, risk assessment becomes a critical tool—not only for identifying threats to heritage assets, but for clarifying how heritage values, practices, and institutions interact with broader socio-environmental systems. This paper situates heritage within these challenges, acknowledging the need for integrated approaches that align conservation practices with climate adaptation, sustainability, and inclusive governance.

We understand heritage as a living process: our legacy from the past, which includes social and ecological systems, within which we live today and safeguard for future generations. This process encompasses not only the protection, conservation, and management of evidence and practices, but also the diverse ways in which communities imbue places, sites, and collections with cultural importance, whether under formal or customary management. Heritage in this sense includes a wide variety of place-making forms and practices that interact with climate and related policies in diverse ways (UNESCO World Heritage Convention, 2023). It is usually differentiated according to typologies, ranging from tangible heritage assets (e.g., historical monuments, buildings, artefacts), intangible heritage elements (e.g., crafts, rituals, oral histories, building practices), and heritage places (the broader socio-ecological elements of a landscape), with each carrying different temporalities, spatial dimensions, and actors’ networks. This is what makes their relationship to scale fundamentally distinct.

At the core of these relationships is the concept of ‘scale’: our aim is to disentangle uses and understanding of ‘scale’ in climate change risk assessments for heritage. To support this investigation, we leverage an established framework, acknowledging that there are relevant scales underpinning policy and management, measurements and observation, and the phenomena (environmental, social, cultural, and political) involved in the risks. We demonstrate how mismatches arise between them and recommend ways to address them within climate change and heritage risk assessment.

### Risk management and climate change adaptation for heritage

Risk management for heritage in the context of climate change is complex, particularly as it is challenging to disentangle the vulnerabilities of objects, traditions, and place and their interactions (Crowley et al. 2022; Fatorić and Seekamp 2017; Sesana et al. 2021). Conventional approaches to heritage conservation, including adaptation, are focused on minimizing the degradation of authenticity or integrity because—unlike infrastructure that can be replaced or upgraded—heritage is irreplaceable, context-specific, and deeply tied to community identity (Holtorf 2018; Seekamp and Jo 2020; UNESCO, 2021).

Heritage requires systematic and scale-aware understanding as it is not confined to discrete sites, categories, or moments in time. Heritage emerges through relations among places, practices, meanings, environments, and communities across multiple spatial and temporal scales (Byrne 2008; Harvey 2015). Furthermore, the scale at which heritage is valued and experienced often differs from the scale at which it is formally managed, creating a disconnect that can preserve physical fabric while overlooking cultural meanings and relationships. Yet, the dominant ‘nature versus culture’ dichotomy has constrained approaches to heritage and climate adaptation, artificially separating ecological and cultural values (Baram 2024). This framing creates false divisions between natural and cultural heritage, sites and landscapes, tangible and intangible practices, and past, present, and future (Baram 2024; Harrison 2015; Smith 2006). Heritage is inherently transboundary, spanning places, time, spaces, and practices rather than being confined to discrete ‘sites’ that can be neatly bounded and managed Accordingly, heritage should not be approached through discrete tasks or reductionist categories alone, but through integrative frameworks that recognise it as a relational, living process shaped by systems-level interactions between people, place, and environment (Harrison et al. 2020).

The diversity of spatial and temporal considerations introduce further complexity for transformative change. Climate change adaptation frameworks for cultural heritage have often emphasized near-term technical fixes based on static understandings of heritage places (Fatorić and Seekamp 2017; Phillips 2015). This narrow scope obscures how heritage significance unfolds across temporal scales, for example across generations, and spatial scales, such as significance changes from material to site to collection of sites. This leads to fragmented decision making that fails to anticipate compounding risks or long-term trajectories (Deubelli and Mechler 2021). Scale mismatches should therefore not only be seen as barriers but also as diagnostic indicators of deeper epistemological divides—for instance, between European modern conservation paradigms that privilege material authenticity and Indigenous or community ontologies that not only emphasize continuity of practice and relational place-making but also embrace change (Holtorf and Högberg 2020; Whyte 2018). For example, UNESCO reporting on Historic Cairo has raised concern over a tendency toward ‘complete restoration’, favouring instead the conservation of signs of age and patina (UNESCO World Heritage Committee 2001), whereas studies of Kathmandu Valley and Patan Durbar Square describe heritage as a living fabric, where continued use, community connection, and the replication of damaged sacred objects may be prioritised over fixed material authenticity (Haselberger and Krist 2022). Recognizing these mismatches opens pathways to rethink what counts as knowledge or evidence in heritage risk assessment and to embed anticipatory, intergenerational governance in adaptation strategies.

### Current understanding of ‘scale’ in climate change risk assessments for heritage

Current understandings of ‘scale’ in climate change risk assessments for heritage have developed heterogeneously. Efforts have focused on hazards and exposure, often with global or regional remit (Bonazza et al. 2021; Chen et al. 2025; Garrote et al. 2020; Moreno et al. 2022). This has advanced knowledge of climate change risks for heritage at a broad spatial scale, but it only partially addresses the broader components of risk assessment (Thomas et al. 2025). Other determinants, such as vulnerability, including adaptive capacity, tend to be considered at much finer scales, if at all, and their connection to global or regional assessments remains underdeveloped. Moreover, temporal and organisational aspects of scale are frequently implicit, leaving little clarity on how they align with spatial analyses. This creates potential mismatches, for instance when exposure is mapped globally but site-level decision-makers require detailed data for planning and adaptation (Chen et al. 2025).

In the state of the art, climate risk assessments for heritage study ‘scale’ most visibly across relevant spatial and temporal dimensions. Spatially, hazard mapping is often conducted at the regional or territorial level, drawing on climate models to identify risk patterns (Bonazza et al. 2021; Chen et al. 2025; Garrote et al. 2020; Moreno et al. 2022). Vulnerability, however, is more commonly assessed at local or building remit, where material properties and performance and site use and management can be examined in detail (Gandini et al. 2021). Increasingly, assessments also adopt a landscape perspective, situating heritage within broader historic and environmental settings (Cook et al. 2021; Ginzarly et al., 2024), or expand to national portfolios for prioritising resources across large estates (Bonazza and Sardella 2023). Temporally, studies combine near-term horizons (e.g., 2030 or 2050) with longer projections (to 2100), helping distinguish immediate pressures from long-term risks (Bonazza and Sardella 2023; Cook et al. 2021; Daly et al. 2021).

Other fields show why scale is central yet difficult to handle. Geography and ecology treat scale as relational, where processes visible at one level may disappear at another (Chen et al. 2025; Maciejewski et al. 2015). Governance and planning research highlights ‘scalar mismatches’, where administrative boundaries rarely align with the spatial or temporal extent of climate risks (Marin et al. 2024; Weinger 2025). Heritage illustrates these challenges clearly: a flood may damage a local monument, but its causes and consequences are shaped by river-basin dynamics and regional governance, and wider pattern s of social and environmental change, requiring coordination across municipal, regional, national and international authorities (Lockwood 2013; Papathoma-Köhle et al. 2016). This is further complicated by the interdisciplinary nature of heritage research and practice, spanning architects, archaeologists, engineers, conservation biologists, historians, and community actors, each of whom conceptualizes scale differently (Sesana et al. 2020). Effective risk assessment must therefore account for the systemic nature of heritage and associated values, as climate vulnerabilities and management strategies vary across materials, practices, places, and governance contexts (Fatorić and Seekamp 2017 a; Harrison et al. 2020; Deubelli and Mechler 2021).Implementation of risk assessment also reveals difficulties. The established IPCC risk model –– incorporating hazard, exposure, vulnerability, and response – does not specify the scale(s) at which each determinant should be assessed (Intergovernmental Panel On Climate Change (Ipcc) 2023b). In practice, hazards may be measured using remote-sensing or national datasets (Aboulnaga et al. 2024; Moreno et al. 2022), vulnerabilities assessed at the community level (Ginzarly et al. 2024), and policy responses framed nationally or internationally (Burke et al. 2019). When these scales are implicitly assumed to be coherent, potential mismatches emerge: global climate data often prove unusable for site-level managers, while national strategies overlook the vulnerabilities identified locally (Daly et al. 2021).

Despite advances, challenges related to scale in cultural heritage risk assessment and modelling are prominent across the field and sector, highlighting complexities in methodology, data availability, and practical application. Downscaling climate data from global or regional models to site conditions remains problematic, as hazard maps lack the granularity needed for local deterioration processes (Julià and Ferreira 2021). At national or estate levels, assessments are constrained by incomplete or inconsistent data, particularly on conservation states and materials (Arosio et al. 2024). Comparability across contexts is weakened by the absence of international standards for hazard classification and vulnerability indices. Long-term monitoring for heritage (on the scale of climate, e.g. 10 years), let alone those for climate *change* (comparison of 10- or 30-year periods) is scarce, making it difficult to separate climate change impacts from natural variability. Future projections remain uncertain, with wide margins of error and poorly defined correlations between hazards and vulnerabilities (Cook et al. 2021). Finally, socio-economic and cultural factors are often sidelined, leaving assessments overly reliant on physical parameters and less able to capture the holistic vulnerability of heritage (Quesada-Ganuza et al. 2021).

The need for a structured approach to represent scale in assessing the risks of climate change is not unique to heritage. Research on climate change has long examined the challenges of aligning temporal and spatial scales in decision-making (Cook et al. 2021). Yet heritage presents distinctive difficulties: its temporal significance spans centuries, its values are plural and contested, and its governance is fragmented across misaligned administrative and epistemic scales (Burke et al. 2019; Ginzarly et al. 2024). These characteristics make heritage especially vulnerable to scale-related incongruences; yet also provide opportunities to inform how climate change presents challenges for other value-driven sectors.

Assessing the risks of climate change for heritage therefore requires attention to observation, measurement and operational aspects of scale. A more robust approach is to explicitly characterise which scales are relevant, how they interact, and where mismatches occur. Without this, assessments will be fragmented, leading to ineffective adaptation strategies. To address these complexities, the next section introduces a framework that distinguishes four meanings of scale. This provides a clearer conceptual basis for assessing climate risks to heritage and for navigating the unique multi-scalar challenges it presents.

### A framework: four meanings of scale

In discussing climate change risk assessment for heritage, we adopt an established framework, *Four Meanings of Scale* (Sheppard and McMaster 2004) as a lens through which to examine this challenge. This framework distinguishes between cartographic, observational, measurement, and operational scales. Cartographic scale is the map ratio between the fabric (e.g., paper or screen) distance and the real-world distance. While it is a fundamental geographic concept, it is not critical to the focus of this study. Since the advent of digital mapping, the cartographic scale in risk assessment can be instantly rescaled (Christopherson 2013).

Here we adopt observational, measurement, and operational scales as the core concepts in describing the challenges of scale in climate change risk assessment for heritage. Scale is not simply a fixed, hierarchical structure of nested levels; rather, it is relational and contingent, emerging through dynamic interactions among actors, processes, policies, and decision-making systems. Because of this dependency, a pattern seen at one scale can look very different when the observation window widens or narrows (Huang et al. 2023). While we acknowledge the important of intra-scale analysis or comparison (for example, observing the same phenomenon at two spatial scales), herein we focus on pairwise intersections between observational, measurement, and operational scales (Table 1) and their dependencies.

**Table 1:** Three scales adopted from Sheppard and McMaster (2004) to discuss the challenges of scale in climate change risk assessment for heritage. The table summarises the roles of the three scales in geography and heritage risk assessment contexts. It highlights how each scale relates to spatial and temporal dimensions, and how risks are framed, measured, and understood

Scale	Definition (Sheppard and McMaster 2004)	Example in CCRA for heritage
Observational (study extent)	The spatial and temporal boundaries selected for a study, i.e., study extent.	Spatially, the study extent might be the extent of a managed area (such as a site), pertain to a particular region, or be defined by the extent of a particular cultural practice not aligned with any specific administrative boundaries this can follow legal or administrative boundaries yet also include cultural links that extend them. Temporally, a risk assessment might be aiming to inform adaptation planning in the next 10 years, or project what the most significant risks will be in two generations’ time
Measurement (grain or resolution)	The smallest distinguishable unit of data, such as a map pixel or the sampling interval during a survey. It determines the level of detail.	Spatially, the measurement scale is determined by what is trying to be captured but is generally defined by resolution. For example, you might map the quality of a building in an annotated element-by-element condition survey, or give the entire building an overall condition score to be compared to other buildings or a wider site or urban context. Measurements can include a vast range of temporal scales and is relevant to all four determinants of CC/heritage risk (hazard, exposure, vulnerability, response). Environmental monitoring might capture conditions of temperature and humidity every 1–5 min. Conversely, the same parameters might be captured by manual inspection of devices once daily, for example at midday. Climate change information is often found at annual, decadal, or ‘climate’ (30 years) levels.
Operational (processes)	The spatial and temporal frames and/or extents at which a process occurs (natural and social), independent of how it is observed or measured.	Spatially, the scale of deterioration processes might occur within the microstructure of porous building materials. On a much larger spatial scale, jet streams are fast, narrow air currents within the atmosphere; their dynamics are determined by global climate interactions, and yet have implications for local climates. The operational scale also includes social, economic, and cultural processes and activities, and the scales on which they operate, for example nomadic lifestyles in Mongolia (Upton 2010). Temporally it is the rhythm of the process: physically, decades of salt crystallisation can slowly weaken masonry yet a five-minute cloudburst can cause significant material loss; from a human perspective, the 20-year rebuilding cycle of the Ise Grand Shrine in Japan underpins the process of management (Sand 2015).

To strengthen the integration of scale into climate risk methodologies, we argue that risk assessment must be understood as a component of broader risk management, not as a standalone diagnostic tool. This reframing positions risk assessment as a dynamic process that informs decision-making, governance, and management strategies. Rather than assuming that scale mismatches inherently lead to failure, we propose that they can serve as diagnostic indicators—revealing underlying disconnects in values, priorities, and institutional arrangements. These challenges are not new; however, climate change introduces new urgency and complexity, demanding shifts in how measurement is conducted and how environmental change is conceptualised. The sections that follow introduce each type of scales and their relevance to heritage climate change risk assessment; interrogate how the mismatches between these scales leads to oversights for the field; provides recommendations to address each of these misalignments; and finally concludes on future avenues of research for the sector.

## The scales

### Observational

The observational scale includes the spatial and temporal boundaries of a climate change risk assessment. Observational scale is not limited to the ‘scope’ of a risk assessment, but to the framework within which that scope is justified—based on spatial extent, temporal framing, and decision-making relevance. It establishes ‘where’, ‘for whom’, ‘why’, and ‘for which decisions’, so that the analysis is both feasible and aligned with the questions posed. This includes deciding which forms of heritage fall within the remit, the relevant timescales, and the prioritisation of decision making. Therefore, the observational scale defines the objective of the risk assessment, positioning broad-scale climate information and site-specific evidence as complementary sources of knowledge for heritage management. The observational scale is thus highly context specific, and co-produced by multiple actors—heritage professionals, communities, rights-holders, policymakers, and where relevant Indigenous knowledge holders. The decision-making relevance is not simply a technical criterion but emerges from plural values, contested priorities, and governance arrangements.

Heritage sites are defined through diverse frameworks—administrative boundaries, ownership, or designation categories—yet these do not always align with the scales required for climate risk assessment. Immovable heritage is inherently place-based and often managed as a ‘site’ with a defined location and boundary. These boundaries cannot accurately capture the full exposure of a site to climate change as site extents are usually not based on ecological processes, but rather are determined by historical significance, and contemporary administrative and managerial concerns. Yet even where heritage is materially fixed, the relevant observational scale may extend beyond site boundaries because the communities through which it is valued, use, or governed may be spatially dispersed, mobile, or only partially co-located with the site itself.

The features and characteristics of heritage that are valued, i.e., significance, determine the elements of focus within the observational scale. Heritage value is complex: it can be broadly understood as value ascribed to features and characteristics beyond their mere utility. Therefore, observational scale must account for whether the objective is to maintain material integrity or to sustain heritage through continuity, renewal, or adaptation, recognising that different conservation traditions place different weight on material fabric, continued use, and change over time (Poulios 2014). Observational scale is therefore shaped not only by the spatial extent of a site, but also by the collectivities through which significance is defined, contested, and sustained.

In a climate change context, risks may (i) directly damage valued features and/or characteristics; (ii) indirectly disrupt the operations, infrastructure, or services that enable access, education, and management, or negatively impact emotional, symbolic, and intergenerational meanings that community members associate with heritage, or continued use; (iii) affect associated communities whose livelihoods or habitation depend on a heritage site; or (iv) influence emotional, symbolic, and intergenerational meanings that community members associate with heritage (Henderson and Seekamp 2018). A risk assessment must decide which of these, if not all, lie within scope of the observational scale.

In many cases, the primary concern will be adverse consequences for valued features and for the operations and services that sustain access, use, and management. Assessing climate change risks to these must be underpinned by a robust and current understanding of the values associated with a particular instance of heritage, introducing a challenge for the temporal aspects of the observational scale. Heritage that has been designated in the past may not hold the same values in perpetuity. If a climate change risk assessment is done based on inaccurate assessments of associated values, the most pressing adverse consequences (impacts) may not be identified or assessed.

Climate change risk measurements therefore span temporal dimensions that range from urgent remedial action to long-term, inter-generational stewardship, while acknowledging that future generations may prioritise different values. Because every risk assessment has an implicit temporal boundary—how long the identified values should endure and when interventions must occur—the spatial boundary selected for an observational scale is inseparable from the temporal scale of heritage management decisions. This is further complicated by the variation in expected lifetimes and perseverance of heritage across stakeholders involved in heritage management (Dillon et al. 2012), and planning frameworks.

### Measurement

Measurement scale—often called resolution or ‘grain’—is the size of the smallest distinct element that can be recorded about a phenomenon (Tobler 1987). In digital form, it might be a single pixel in a satellite image or the five-minute interval of an environmental datalogger. Unlike cartographic scale, which changes when one zooms in or out on a map, resolution is fixed once the data are collected; enlarging the display does not reveal new detail (Quijas and Balvanera 2013).

High-resolution, in-situ measurements for heritage focus on spatial granularity relevant to the context, using tools like climate sensors in a museum display case to three-dimensional modelling and photogrammetry to capture localized threats such as humidity fluctuations and material deterioration (Rosina et al. 2019). Finite monitoring campaigns provide time-bound insights, such as a single dive to inspect an historic shipwreck, balancing resource constraints with targeted data collection.

On the other hand, global or continental datasets afford broad coverage (Figliola et al. 2025). CMIP6 climate ensembles (Intergovernmental Panel on Climate Change (Ipcc), 2023), ERA5 Reanalysis (Hersbach et al. 2020), and medium-resolution satellite sensors (250 m to 1 km resolution) (Hao et al. 2024), offer cross-regional comparisons and help the identification of high-risk heritage sites. Rather than a competing choice, these scales serve complementary roles: broad-scale datasets identify the wider climate patterns that drive hazards, while local measurements and site-specific evidence refine, validate, or supplement those patterns to understand how impacts are experienced on the ground.

Resolution need not be static in either space or time for measuring climate change risks for heritage. For instance, the ISO 15,927 standard for semi-empirical assessments of wind-driven rain hazards for façade requires hourly or three-hourly data resolution for precipitation, wind speed, and wind direction (International Organization for Standardization, 2009). However, it has been demonstrated that these representations correlate very well with similar indicators produced from daily, or even monthly aggregated variables (Pérez-Bella et al. 2025). This principle could be applied to other types of risks: once those relationships are calibrated, varying the sampling interval and spatial density of monitors can increase the scalability of climate change risk assessments.

For intangible heritage, the unit of measurement is rarely a map pixel; it is often an event, tradition, or a practice tied to particular places and seasons. Intangible cultural heritage, which is often associated with Indigenous, local and traditional knowledge, is usually directly related to climate and territories. It is place-based and therefore, climate risks to a place will influence and impact on potential losses and damages to intangible cultural heritage (e.g., agricultural practices where seasonality is shifting (A. P. Smith et al. 2021), fishing traditions when ocean warming impacts marine life (Cheung 2018), traditional crafts reliant on a material threatened by land use change) (Dereso and Seid 2019). Therefore, assessing climate risks to intangible cultural heritage needs to consider these systemic interdependencies.

### Operational

Operational scale refers to the range of spatial and temporal frames at which a process (whether environmental, social, cultural, or economic) actually occurs, regardless of how they are observed or managed. Sayre (2005) defines a useful conceptual distinction between the operational scale and the observational scale: the operational scale is the process’s ontological moment—the level at which it is real in the world—whereas the observational scale is the researcher’s epistemological moment—the level at which it is examined (Sayre 2005).

The impacts of climate change can evolve gradually (over decades or centuries), or be instantaneous and acute (IPCC, 2023a). For example, water infiltration in porous masonry may arise from the interaction of multiple processes: slow rising groundwater, periodic heavy rainfall, and salt transport (Sesana et al. 2021). These factors together may lead to structural weakening through salt crystallisation and expansion (Desarnaud 2024). A wall may endure gradual changes for years, but a single intense storm could precipitate rapid failure (though attributing this to climate change is a nascent field). Such dynamics reveal why operational scale matters: long-term degradation and sudden impacts must be assessed together.

Considerations of the operational scale are equally relevant for intangible heritage. For example, the annual cycles of sourcing, preparing, and applying natural materials that sustain Japan’s traditional wooden-building craftsmanship provide an operational scale for evaluating how climate change could shift work rhythms, material quality, and knowledge transfer (Sand 2015). A single season of extreme heat or heavy rainfall may delay timber harvesting or plaster curing, disrupting an apprenticeship schedule dependent on those timings. Repeated disruptions over many years could shift the craftsmanship convention. Understanding how climate change is shifting these patterns is central to assessing the risks. The operational scale is independent of how climate risk is measured or observed, rather it is the processes that underpin the risks to be managed (Richards and Brimblecombe 2025).

## Interscale interactions within climate change and climate risk assessment for heritage

Hereafter, we use ‘mismatch’ to describe when one aspect of scale within the context of risk assessment does not match at least one other. This concept is derived from human geography such as the Spatial Mismatch Theory (Gobillon et al. 2007; Wang et al. 2022). Built upon this concept, Sheppard and McMaster (2004) argue that mismatches emerge when whenever observational extent, measurement grain, and operational framework are disconnected. For instance, comparing century-long sea-level projections with a single year of on-site humidity measurements or applying a national risk assessment to a single site without considering the local context, are considered mismatches.

The subsections below describe how mismatches between information (measurement scale), processes (operational scale), and decisions (observational scale) emerge as key challenges in the context of climate change risk assessment for heritage.

**Fig. 1:**
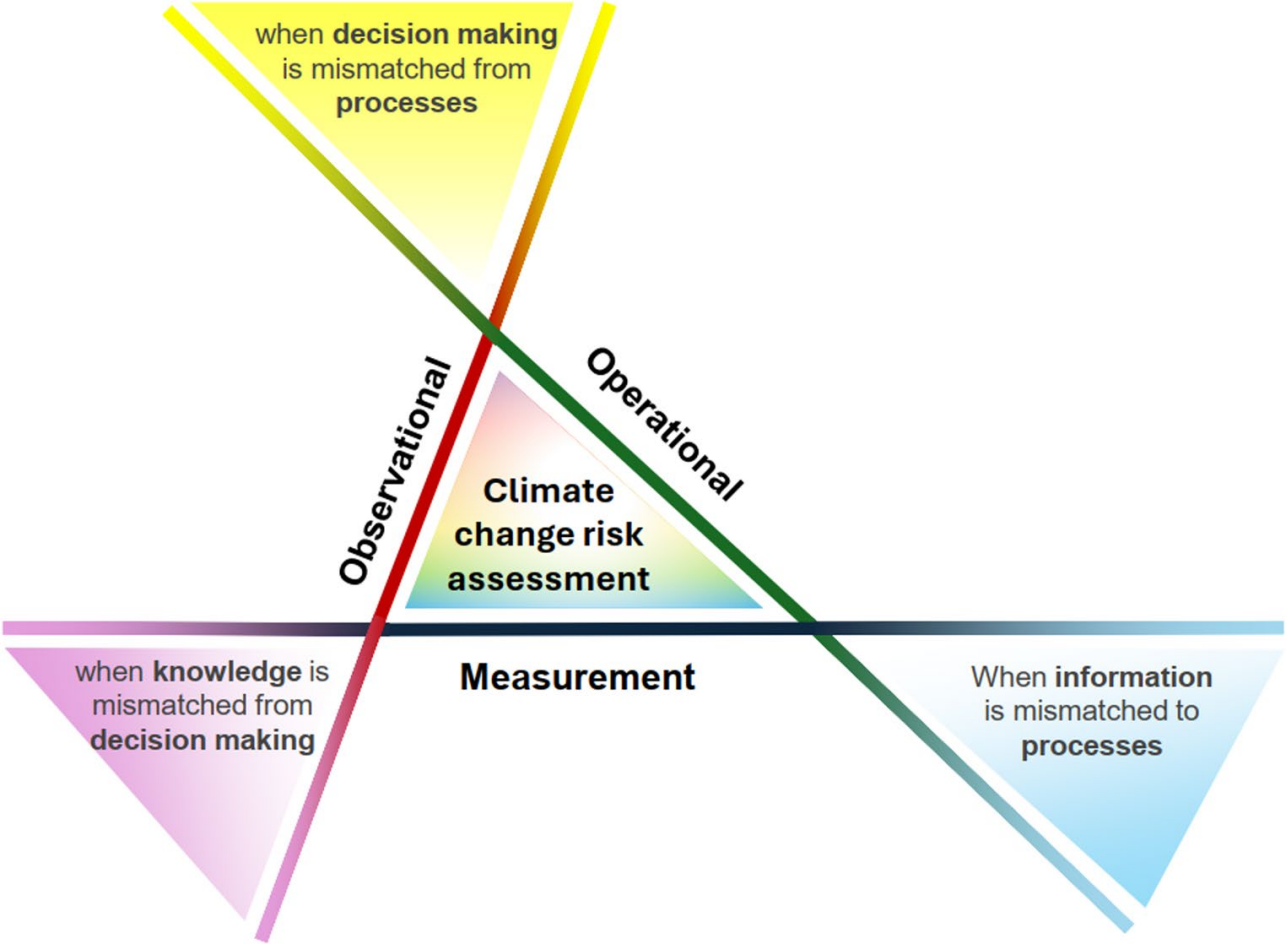
A visual representation of climate change risk assessment (CCRA) at the intersection of measurement, observation, and operational scales, showing the pairwise mismatches between them.

**Fig. 2:**
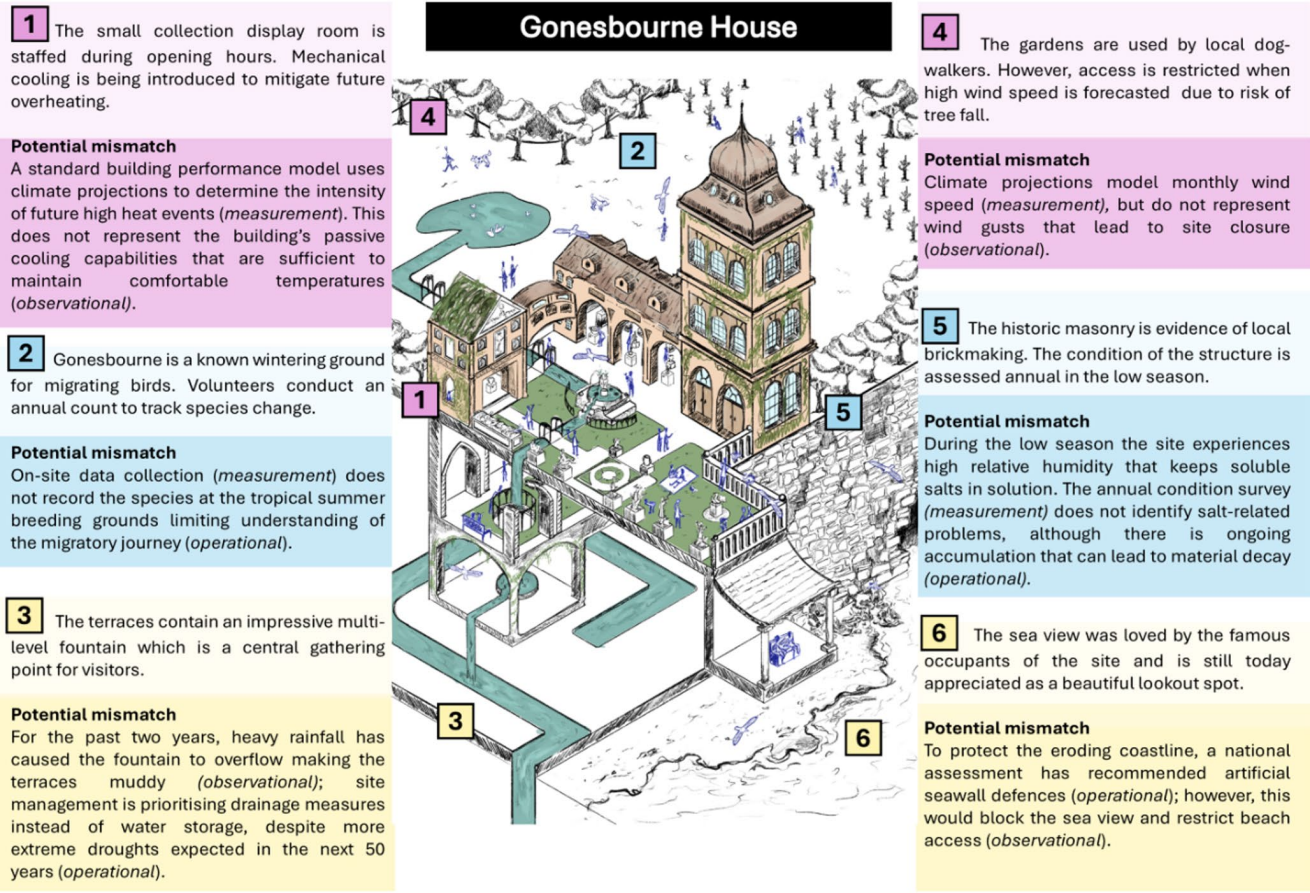
The framework proposed herein applied to a fictitious example of Gonesbourne House to demonstrate the potential mismatches of scale. The site is a managed and visited heritage place, comprised of buildings, collection items presented within them, and a landscape and biodiversity context in which it is situated

Climate change risk assessment lies at the intersection of the observational, measurement, and operational scales, but becomes very complex when considered all together. Herein we study pairwise intersections (Fig. 1) to isolate specific potential mismatches and their implications. These pairwise intersections are each described in detail in the subsequent sections and also demonstrated by a fictitious example of a heritage place (Fig. 2).

### When information and decision making are mismatched (observational and measurement scales)

#### Spatial aspects

This mismatch arises when measurement or granularity of information, as represented by knowledge, reports, data or tools, is mismatched to the observational remit or extent, such as a site boundary, a particular area or feature of significance, or decision-making context.

One of the main causes of this mismatch is that our understanding of future climate conditions is primarily informed by large-scale global projections. These are often produced at continental or regional scales, while heritage managers need highly localised information on hazards to inform decision making. In this scenario, there is a potential for localised conditions not to be accurately represented by these larger-scale projections.

In contrast, at the national scale or for a similarly-scaled administrative boundary, assessing hazards for heritage can more reasonably rely on large-scale datasets and technologies such as LiDAR, remote sensing (Cuca et al. 2023), and climate projections. This is because they will generally be more closely matched in scale to national heritage inventories or databases. Of course, the scale of the study extent needs to be considered relative to the scale of the boundary: a global climate model at 100–200 km square resolution will represent the national-scale contrasts for Canada (the second-largest country by total area) far better than for Malta (which is about 20 km from end to end). In the latter case, the entire country is likely to be within a single grid cell, which implies that the whole country will experience exactly equivalent climates in future. These tools enable the identification of broad patterns of risk for a portfolio of heritage and the prioritisation of resources for areas facing the most significant hazards.

However, even some of the finer-resolution climate models—such as ERA5, with a horizontal resolution of approximately 31 km—remain too coarse relative to the spatial scale of most heritage (Hersbach et al. 2020). Recent advances in satellite remote sensing have demonstrated that high-resolution optical and imagery (10–30 m, and in some case meter-scale) can support site-level damage detection and post-event assessment for cultural heritage, particularly for sudden hazards such as earthquakes or landslides (Cuca et al. 2023; Mazzanti et al. 2022). However, these satellite applications are primarily suited to identifying surface damage or structural change, rather than capturing the microclimatic, material, or indoor environmental processes that drive heritage-specific impacts. Similarly, assessing the impact of large-scale atmospheric processes like jet stream variability on material longevity (e.g., environmental conditions and implications for museum collections) risks misrepresenting microclimatic drivers such as indoor humidity or building envelope performance. Coarse-resolution climate data cannot capture these localized conditions, leading to uncertainties for heritage site-level risks.

Further challenges arise when local measurements and knowledge are incorporated into wider-scale analysis. Condition surveys, vulnerability assessments, and community inputs are essential because sensitivity and adaptive capacity are closely tied to site condition, use, significance, and the relationships between community members and heritage (McCreary et al. 2019). For example, Peek et al. (2022) assessed coastal heritage across more than 40 US National Park Service units using regional exposure indicators alongside site-level measures of damage potential, condition, historical damage, and protective engineering. While such approaches can identify broad differences in exposure, vulnerability remains strongly shaped by local social, cultural, and economic contexts, and by the expertise of site managers and knowledge holders. As a result, broad-scale climate data may indicate the potential for risk, but often miss the site-specific variables and experiential knowledge needed to show how that risk is experienced and managed at the site.

#### Temporal aspects

Temporal mismatches in climate risk assessment for heritage arise when the timescales of measurement (e.g., climate projections, expert knowledge) and the timescales involved in decision making are misaligned. These discrepancies can obscure long-term vulnerabilities or overemphasize short-term events, complicating efforts to plan for intergenerational stewardship and adaptation. Many instances of heritage have existed for millennia. Yet most climate projections used in practice extend only to 2100, the standard end point for many contemporary climate scenarios and assessments (IPCC, 2023a), which captures only a small fraction of their past, present, and future existence. This temporal limitation of climate models is also compounded by the mismatch between the resolution of climate data and the processual timescales of heritage change. For instance, daily or monthly model outputs are often aggregated to produce decadal averages, which can obscure the extreme events that may trigger tipping points in structural integrity or disrupt intergenerational knowledge transmission. Conversely, short-term assessments (e.g., one-year humidity monitoring) may miss cumulative, slow-onset impacts. This challenge calls for approaches that can respond to the deep time of heritage and the long tail of climate uncertainty, such as a narrative storyline approach, multiple scenario planning, and imagining heritage climate futures (Riesto et al. 2022).

The use of the term living heritage (often used alongside intangible cultural heritage) aims to foreground the generational aspect of heritage: that types of heritage must be able to be both inherited and passed down to function (Consultation on the 2003 UNESCO Convention for the Safeguarding of the Intangible Cultural Heritage, 2023; Nic Craith et al. 2018). Each generation thus has its own relationship to its heritage, with valued aspects shifting across the years. This can create decision-making paralysis as 2100 (about 75 years from time of publication) may be the reality of two or three future generations likely with different connections to their living heritage.

### When processes and decision making are mismatched (operational and observational scales)

#### Spatial aspects

A critical spatial mismatch occurs when the operational scale of decision-making or intervention does not align with the observational scale at which heritage is assessed or for which decisions are being made. This mismatch is bidirectional: while large-scale observations—such as national or global warming trends—often prompt hyperlocal interventions, this pattern is so common it is almost ubiquitous. Conversely, large-scale interventions that affect local heritage are less visible but arguably more consequential, as they can reshape site conditions, meanings, and management priorities in ways that are difficult to anticipate. These dynamics highlight the need to examine not only the scale at which risks are observed but also the scale at which responses are implemented, ensuring that adaptation strategies do not inadvertently create new vulnerabilities.

Heritage sites and their immediate surroundings are often managed as a ‘site’, which can be overlooked when intervention is implemented at larger operational scales. Regional irrigation projects, for example, are designed to address agricultural needs, might alter local hydrological conditions. This change at the operational scale could lead to unforeseen consequences at a heritage site within, such as the increased salt ingress and accelerated deterioration that previously did not occur. Similarly, coastal management strategies, such as implementing sea walls or beach nourishment at a regional scale can sever a heritage site’s intrinsic connection to the sea or alter local sediment dynamics, impacting its integrity or setting—and potentially having off-site impacts that threaten stability of the greater socio-ecological systems. For example, at Hurst Castle in England, coastal defences implemented elsewhere have interrupted natural shingle replenishment, leading to increased vulnerability of the site (Lankester and Knight 2024).

Moreover, spatial mismatches can extend beyond the site itself. Jurjonas et al. (2020) argue for climate justice considerations in coastal zone management, noting that interventions must account for shared cultural landscapes and commons that transcend administrative boundaries. This is particularly relevant for heritage that is relational or transboundary in nature—such as pilgrimage routes, maritime heritage, or diasporic cultural practices—where the significance of place is distributed across multiple jurisdictions and communities. These mismatches may also be social, insofar as different collectivises can relate to the same heritage through distinct spatial attachments, or claims that are captured by material- or object-based understandings of heritage, or by administrative boundaries alone. When decision-making frameworks are confined to narrow administrative units, they risk excluding stakeholders who hold cultural, historical, or spiritual connections to the heritage, thereby undermining inclusive governance and equitable adaptation.

#### Temporal aspects

A temporal mismatch occurs when the timescales at which processes occur are mismatched to those at which decisions are made about how to manage them. This is particularly relevant in heritage contexts where long-term, cumulative processes—such as material decay or knowledge erosion—may trigger sudden impacts, yet decision-making frameworks often operate on short-term cycles. For instance, annual freeze–thaw cycles can suddenly be disrupted leading to the collapse of soil structures impacting buried archaeology (Grossi et al. 2007). The challenge lies in aligning proactive operational measures with these slowly developing observational trends before critical thresholds are reached.

Risk assessments often rely heavily on future projections while overlooking past and ongoing climate dynamics. Attribution science (Otto 2017) offers a way to address this by quantifying how human influence has already altered the frequency and intensity of extreme weather events relative to a counterfactual climate. Recent work applying attribution methods at World Heritage Sites shows anthropogenic climate change has already added more than a month of extreme heat days per year, a condition that was rare in a preindustrial context, and can inform international policy coordination and site-level adaptation for cultural heritage (Simpson et al. 2022; Zhao et al. 2026).

Another challenge lies in the limitation of contemporary decision making: heritage management must balance immediate operational priorities against long-term, intergenerational responsibilities. For example, when resources are limited, decision-makers may face difficult trade-offs: should they fully preserve the condition of heritage now, or accept partial loss of historic integrity in exchange for site investments that reduce sensitivity to future storm impacts? This is then compounded by the short-term funding cycle of many heritage grants and research projects. In the worst cases, resources are so limited that no action is possible even with the will to enact them.

These tensions are amplified when contrasting Indigenous and local knowledge systems with dominant conservation frameworks. Local and Indigenous traditions often view heritage as dynamic and evolving, shaped by lived experience and adaptation over time. In contrast, operational decision-making frameworks—shaped by international reference documents such as the World Heritage Convention and the Venice Charter—propose the need to establish conservation baselines (International Council on Monuments and Sites (ICOMOS), 1964; UNESCO World Heritage Centre 2025). This mismatch creates a temporal incongruence between the cyclical or flexible timeframes of place-based knowledge and the linear, static timelines embedded in formal (institutional and Western-modern) heritage management systems. However, conservation policies are increasingly evolving to accommodate dynamic change, such as the UNESCO Recommendation on the Historic Urban Landscape (HUL) and emerging climate risk assessment frameworks (Deubelli and Mechler 2021).

### When information and processes are mismatched (operational and measurement scales)

#### Spatial aspects

Challenges arise when the measurements available do not accurately represent the relevant environmental, social, cultural, or economic processes. These incongruities are highlighted in various contexts. General Circulation Models (GCMs), operating at a global measurement scale, may omit localized operational processes and this especially impacts coastal, urban, and mountain heritage. GCMs overlook the urban heat island effect and require downscaling to properly represent urban temperatures (Creutzig et al. 2025; Miniandi et al. 2025). Similarly, challenges occur in coastline analysis where the detail required to understand processes such as coastal erosion or retreat is lost because grid cells are dominated by oceanic physics and fail to represent near-surface conditions (Vousdoukas et al. 2022). Furthermore, the variations of orography, such as areas of steep terrain, are smoothed in climate models which conventionally simulate the near-surface air temperature at 1.5 to 2 m (Zhang et al. 2022). Over the Tibetan Plateau, 25 km regional models still show cold biases that grow with elevation, revealing unresolved peak-level processes (Li et al. 2023). Even finer-scale downscaling (≤ 25 km) cuts some errors yet still struggles with snow physics and sparse summit observations, so important uncertainties remain. This introduces challenges for assessing risks at the national scale for a territory such as China (e.g., (Wang et al. 2022) or Africa where the 50-km CORDEX model loses accuracy once the land rises above about 3 km (Girma et al. 2022). Risk assessments for mountainous, coastal and urban heritage may rely on climate models that cannot accurately represent the relevant environmental processes, leading to missed early warning signals, misallocated adaptation funding, and inappropriately placed adaptation measures, such as coastal defences.

#### Temporal aspects

Temporal mismatches between operational processes and measurement scales occur when the timescales used to monitor or model climate-related hazards do not align with the rhythms or durations of heritage processes and adaptation. These mismatches can lead to misrepresentation of risks, ineffective early-warning systems, and poorly timed interventions. For example, extrapolating from two years of on-site monitoring to predict long-term climate impacts may overlook the potential cumulative impacts of cycles of salt mixtures going in and out of solution, which operates in sub-hourly increments but accumulates damage over decades (Godts et al. 2021). Further, hourly measurements used to assess the potential occurrence of salt crystallisation events that may induce damage to building materials will underrepresent the actual number of potential events.

Selecting criteria for monitoring also presents challenges in the context of this mismatch. These criteria are often based on temporal thresholds or multi-component indicators for on hazard profiles: their frequency and duration, among others—but these may not always be appropriate. Some indicators are based on vulnerability rather than temporal metrics. For instance, at Hurst Castle, managed by English Heritage, one early-warning indicator is the shingle level falling below a critical point, which reflects geomorphological change rather than a time-based trigger. This demonstrates that temporal mismatches are more relevant for some determinants of risk, particularly if they are evaluated or considered in a multideterminant assessment (Thomas et al. 2025)

## Recommendations

This framework for scale, applied in the context of climate change and heritage risk assessment, acknowledges the mismatches between them. This would improve the rigour and quality of risk assessment and subsequent dynamic adaptive planning (Haasnoot et al. 2013). This section provides pragmatic recommendations (Table 2) within the aforementioned framework for how risk assessments of climate change for heritage can actively minimise the impact of potential mismatches on action. The main thrust of these recommendations is that risk assessment methodological design needs to explicitly recognize and reconcile operational–observational–measurement mismatches, and take a more reflexive, yet procedural approach to ‘scale-awareness’ within risk assessments beyond. As well, to recognise risk assessment as part of the broader, ongoing cycle or process of managing climate change risks and adaptation, such as the adoption of iterative approaches, adaptation pathways (Werners et al. 2021), and adequate monitoring

**Table 2:** Summary of each pairwise mismatch alongside recommendations for the field and sector of how they can be mitigated

Mismatch	Scale dimension	Consequence for risk assessment	Recommendations
When information and decision making are mismatched (observational and measurement scales)	Spatial	Site-level heritage risks may be obscured or mischaracterised when coarse-scale climate data fail to capture microclimatic, material, or contextual processes, leading to inappropriate risk prioritisation, uncertain site-specific projections, and potential discounting of local experiential knowledge that is critical for understanding how hazards are expressed in place.	• combine multi-resolution datasets • apply justified spatial downscaling • documentation of the climate models metadata • development of relevant digital skills and increased usability and accessibility of relevant data • validate projections using site-specific indicators (material thresholds, hydrology, geomorphology) • integrate participatory mapping to capture locally significant processes and spaces
	Temporal	Long-term vulnerabilities, cumulative degradation processes, or critical thresholds may be overlooked when climate data aggregation or short monitoring periods obscure extreme events, slow-onset change, or the deep temporal horizons inherent to heritage, complicating intergenerational decision-making and adaptive stewardship.	• employ time-series analysis across historical and projected datasets • supplement averages with seasonal and event-based indicators • use historical baselines and scenario planning to reveal cumulative and threshold effects.
When processes and decision making are mismatched (operational and observational)	Spatial	Adaptation interventions implemented at scales mismatched to how heritage is experienced and valued can generate unintended consequences, including new physical vulnerabilities, loss of cultural meaning, stakeholder exclusion, and contested outcomes that undermine resilience and legitimacy.	• select indicators aligned to operational footprints and decision making (sites, landscapes, routes, communities) • design monitoring to reflect management and use zones • prioritise multi–level stakeholder engagement to target mismatches • overlay measurement data with governance and process boundaries and buffer zones to identify gaps
	Temporal	Short-term operational decision cycles may fail to anticipate long-term, cumulative, or irreversible change, leading to delayed or reactive adaptation, difficult trade-offs between present condition and future resilience, and tensions between institutional conservation timelines and dynamic, place-based temporalities.	• implement adaptive monitoring frameworks that evolve with changing risks and new knowledge • align data collection frequencies with planning horizons • review and update indicators as priorities, risks, and knowledge change. • Engage with place-based adaptation frameworks, incorporating knowledge from local, Indigenous, and intergenerational groups
When information and processes are mismatched (operational and measurement)	Spatial	Risk assessments may produce technically robust but operationally irrelevant outputs when metrics do not align with site boundaries, buffer zones, or patterns of use, resulting in maladaptation, inefficient allocation of resources, and regional assessments that overlook site-specific vulnerability and cultural context.	• map governance scales against operational realitiesuse buffer zones and community perspectives • support multi-level coordination through participatory, cross-sectoral planning mechanisms. • ground-truth results of large-scale climate models, with known localised processes and community-based monitoring
	Temporal	Decisions may be based on incomplete or misleading evidence when monitoring timescales do not reflect the cumulative, episodic, or threshold-based nature of heritage change, weakening early warning capacity and reducing the effectiveness and timing of adaptation actions.	• adopt adaptation pathways and anticipatory governance • develop temporal indicators that reflect cumulative stress and impact • employ monitoring protocols aligned with the speed of heritage damage processes • identify operational thresholds through time-series analysis and historical observations • integrate local, traditional, Indigenous, and intergenerational knowledge to reflect evolving values and generational understandings

Assemblage theory offers a valuable conceptual lens to enhance scale-aware climate change risk assessments for heritage. By viewing heritage and climate risks as emergent from dynamic, multi-component systems (De Landa 2016) —comprising material conditions, social practices, institutional arrangements, and environmental processes—assemblage theory helps move beyond static or siloed understandings of scale. It foregrounds the relational and contingent nature of risk, where mismatches between observational, measurement, and operational scales are not merely technical issues but reflections of deeper epistemic and governance complexities (Guttormsen et al. 2023). Integrating assemblage thinking into risk assessment design encourages more reflexive, adaptive, and inclusive methodologies that better capture the lived realities of heritage and the distributed agency of climate impacts across scales.

## Conclusion

Herein we have applied the observational-measurement-operational scale framework for climate change and heritage risk assessments. We have demonstrated how potential mismatches arise between these, and recommended ways that the impact on heritage climate action can be reduced.

While heritage has long been tasked with stewardship responsibilities, these mandates are increasingly strained by fiscal limitations, particularly as climate change introduces new layers of complexity. Heritage organisations often receive management authority only once assets are already in decline, and each year more heritage ‘comes of age’ to be listed, acquired, and protected. This expanding portfolio, coupled with the anticipatory nature of climate adaptation, places significant pressure on prioritization. This leaves us with big questions: what should be protected at a site? Which sites across a portfolio? What types of heritage, and whose values, will guide these decisions?

These questions are inherently scalar, and the mismatches identified in this paper between observational, measurement, and operational scales underscore the need for decision-making support tools that can operate across these dimensions. Such tools must enable heritage managers, institutions, and communities to navigate trade-offs, allocate resources, and plan adaptively. Without them, fiscal constraints risk compounding epistemic and governance disconnects, leaving heritage vulnerable not only to climate impacts but to systemic neglect. In this sense, heritage also provides a clear case through which broader cross-sector problems of scale, values, and governance in climate risk assessment can be diagnosed. 

## Data Availability

No datasets were generated or analysed during the current study.
